# Irrigated Tip Catheters for Radiofrequency Ablation in Ventricular Tachycardia

**DOI:** 10.1155/2015/389294

**Published:** 2015-01-29

**Authors:** Andreas Müssigbrodt, Matthias Grothoff, Borislav Dinov, Jedrzej Kosiuk, Sergio Richter, Philipp Sommer, Ole A. Breithardt, Sascha Rolf, Andreas Bollmann, Arash Arya, Gerhard Hindricks

**Affiliations:** ^1^Department of Electrophysiology, University Leipzig, Heart Centre, Strümpellstrasse 39, 04289 Leipzig, Germany; ^2^Department of Radiology, University Leipzig, Heart Centre, Strümpellstrasse 39, 04289 Leipzig, Germany

## Abstract

Radiofrequency (RF) ablation with irrigated tip catheters decreases the likelihood of thrombus and char formation and enables the creation of larger lesions. Due to the potential dramatic consequences, the prevention of thromboembolic events is of particular importance for left-sided procedures. Although acute success rates of ventricular tachycardia (VT) ablation are satisfactory, recurrence rate is high. Apart from the progress of the underlying disease, reconduction and the lack of effective transmural lesions play a major role for VT recurrences. This paper reviews principles of lesion formation with radiofrequency and the effect of tip irrigation as well as recent advances in new technology. Potential areas of further development of catheter technology might be the improvement of mapping by better substrate definition and resolution, the introduction of bipolar and multipolar ablation techniques into clinical routine, and the use of alternative sources of energy.

## 1. Background

A thorough understanding of the arrhythmia and the cardiac anatomy is the prerequisite for successful catheter ablation. It also requires technology to enable the accurate positioning of the catheter and to create the right kind of lesion in order to affect the arrhythmia's pathway(s) but not to cause collateral damage.

Direct current (DC) energy was the first and—from the first successful ablation in a human in 1982 until approximately 1989—the most commonly used energy source for performing catheter ablation. The resultant vaporization of blood created a virtual explosion within the heart with a high risk of “collateral damage.” As DC energy was largely limited to the ablation of the His bundle and because of its traumatic nature, DC energy proved to be of limited utility and alternative sources of energy were being sought [1].

Nowadays, radiofrequency (RF) is the source of energy that is being used for catheter ablation in the vast majority of the EP laboratories around the world. RF energy consists of alternating current with a frequency range of 100 to 2000 kHz. RF energy had already been used in operating rooms to cauterize small bleeding vessels. Attaching an RF generator to an electrode catheter placed inside the heart permits the application of RF energy with the creation of a discrete intracardiac lesion.

RF energy is being passed from the tip of the catheter through the patient's circuit. As electrical current is meeting electrical resistance around the catheter—due to Ohm's law—voltage drops with growing distance from the catheter tip, and—due to the law of the maintenance of energy—RF energy is being converted into heat. This process—resistive heating—starts at the same time as the current flows into the tissue and affects the tissue within a few millimetres from the catheter tip. The heat is also being transferred to the surrounding tissue by conduction and radiation and decreases by the fourth power with the distance from the catheter tip. This secondary process occurs later during the ablation pulse and contributes as well to the size of the lesion.

A rise of the tissue temperature above 50 degrees Celsius leads to the denaturation of proteins. The resulting cell damage interrupts the transmission of electrical signals. RF lesion size is a function of RF power delivery and exposure time.

Thermal energy is distributed within the surrounding tissue, but also to the surrounding blood stream and the catheter tip. Thus, the temperature measured by the temperature sensor in the catheter tip indicates only approximately the temperature of the tissue itself. At the electrode interface, RF current preferentially flows into the blood because of its lower impedance as compared to tissue. Due to the different impedances and due to most anatomical situations with only a small part of the catheter tip surface attaching to the tissue, it has been estimated that only 9% of the total power is being delivered to the tissue, that is, 4.5 W at a total power of 50 W for a 4 mm catheter with good (25%) contact [2].

In the early days of RF catheter ablation, coagulation of blood at the catheter tip frequently caused a sudden increase of the impedance and ablation became less effective and was stopped by impedance control. The introduction of temperature control reduced excessive heating of the tissue and the blood, thereby also reducing the occurrence of coagulum formation.

Cooling of the electrode and the tissue by the blood flow reduces excessive heating and increases thereby the size of the lesion. The cooling effect of the blood stream can be enhanced by an increased surface of the catheter tip. The cooling effect could also be enhanced by cooling the blood or by increasing the blood flow, things that are difficult to realize. In contrast, due to hemodynamic reasons, as atrial fibrillation or reduced ventricular function, or due to anatomic reasons, as pouches, trabecular crevasses, or narrow vessels like branches of the coronary sinus, low blood flow occurs. Thus, the cooling effect of blood flow is not always readily available. Active cooling of the catheter tip, however, is technically possible and was approved in the United States in 1999. This technology uses room temperature saline to reduce the electrode tip temperature during RF ablation. It has been developed to achieve an increased lesion size and a decreased likelihood of thrombus and char formation.

## 2. Types of Ablation Catheters

In contrast to the ablation of common supraventricular arrhythmias such as atrioventricular nodal reentry tachycardia (AVNRT) or Wolff-Parkinson-White (WPW) syndrome, where ablation of rather small sites yields excellent results, ablation of ventricular tachycardia (VT) requires more extensive lesions for effective results. In up to 50% of patients with nonidiopathic VT, RF current delivered through a conventional electrode catheter could not destroy the arrhythmogenic substrate [3, 4]. Different technologies have been developed for RF ablation to increase the efficacy of the ablation, while trying to minimize risks for complications. These different technologies include increased electrode size for enhanced passive cooling via the blood flow and active cooling of the electrode through saline fluid cooling either internally (closed-loop) or externally (open-loop) [5].

At present, four major types of RF ablation catheters are available for clinical use:

1. standard 4 mm tip catheters,
2. large 8–10 mm tip catheters,
3. open-loop irrigated tip catheters,
4. closed-loop irrigated tip catheters.

Nonirrigated 4 mm catheters and nonirrigated 8 mm catheters have been compared in patients with VT of different origin and different aetiology, using remote magnetic navigation technology. In patients with structural heart disease, success was achieved in 22% with the 4 mm catheter tip and in 59% with the 8 mm catheter tip. In patients without structural heart disease and right ventricular outflow origin, success was achieved in 85% with the 4 mm catheter tip and in 87% with the 8 mm catheter tip [6]. Thus, larger catheters expose a greater surface to the blood and may therefore create more efficient lesions by the indirect cooling effect of the blood stream. However, larger electrode tips also have disadvantages in comparison to standard tip size: varying tissue contact with the potential decrease of power transfer, reduced impedance feedback with possible excessive tissue heating, and impaired resolution of the local electrogram [2]. Irrigation of catheters should overcome these limitations.

As power delivery is often limited by high temperatures at the electrode-tissue interface in nonirrigated systems, active tip-electrode cooling in open-loop irrigated tip catheters increases power delivery. It has been demonstrated that the maximal temperature generated by cooled RF application will be several millimetres distant from the electrode-tissue interface, thereby creating a larger lesion depth, width, and volume [7]. Active tip-electrode cooling might also decrease the risk for thrombus and char formation. Due to the dramatic consequences of thromboembolic events, this is of particular importance for procedures within the left heart chambers.

**Figure 1:**
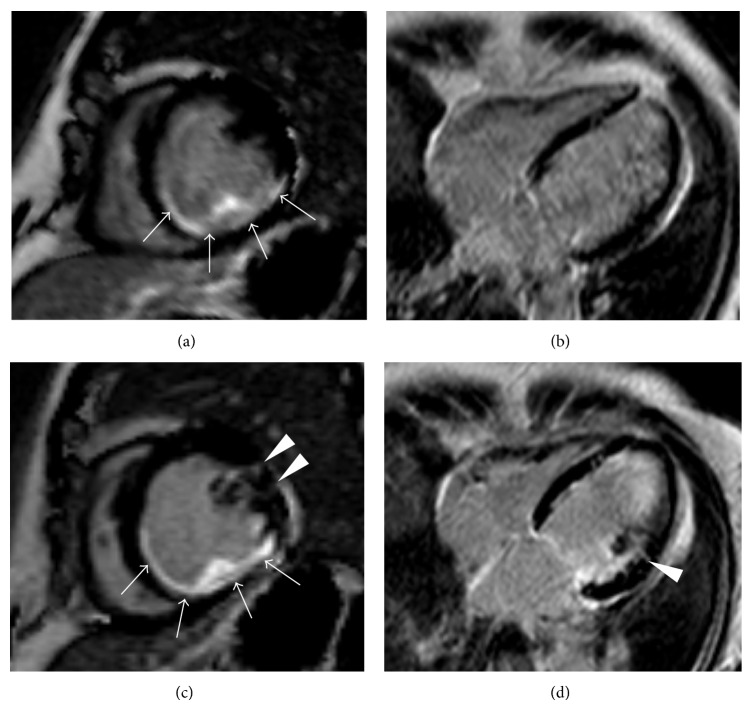
Phase sensitive inversion recovery (PSIR) late Gadolinium enhancement (LGE) images in short axis (a; c) and 4-chamber view orientation (b; d). The first row (a; b) shows the images before ablation of ventricular tachycardia and premature ventricular contractions at the midventricular lateral wall. There is a large area of fibrosis at the inferior and inferolateral wall (arrows) from previous ablation. Images 1 day after ablation (c; d) show small linear transmural LGE (arrowheads) which is interpreted as postablational necrosis.

While the clinical efficacy of open-irrigated RF catheters is well established in clinical practice, there have been studies demonstrating variable lesion depth, shape, and location based on the irrigant or saline “cloud” [5]. This feature of open irrigation is mainly dependent on local factors such as catheter position and tissue contact and direction and magnitude of blood flow. Closed-loop irrigated RF ablation provides tip cooling by internally circulating fluid and has been demonstrated to provide similar advantages in obtaining lesion depth while providing consistent lesion size that is not dependent on these factors. Open irrigation systems have nevertheless demonstrated greater interface cooling with lower incidence of both thrombus formation and steam pops than seen with closed-loop irrigated RF ablation [8]. Lesion formation after irrigated tip catheter ablation of VT and PVC (premature ventricular contractions) can be visualized by magnetic resonance imaging (Figure 1).

## 3. Importance of Catheter Contact and Contact Force

The aim of the ablation is to create a lesion with a sufficient size but without perforating cardiac walls. Catheter ablation can therefore also be considered as a limited destruction, balancing between too little and too much.

If 25% of the catheter tip surface has contact with the underlying tissue, the effective power delivery to the underlying tissue has been calculated to be approximately 9%. With more or less tissue contact, the relation between efficient power delivery to the tissue and losses in the blood pool may vary dramatically. With 50% catheter tip surface-tissue contact, approximately 21% of the power can be delivered instead of 9% [2].

The deeper lesions created in an animal model with high catheter contact pressure are therefore most likely caused by a greater power transmission to the tissue [9].

The mechanical forces required to perforate cardiac walls with an ablation catheter were also evaluated in an animal model. The force threshold for mechanical perforation in the porcine heart was found to be lower for right- compared with left-sided chambers, and also lower through recently created RF lesions compared with nonablated tissue. Left ventricular perforation was achieved more rapidly with the ablation catheter in a sheath despite the same perforation force because the sheath prevents catheter buckling [10].

Lesion size correlates with contact force and contact time. The force-time integral has been evaluated in an in vitro model regarding the lesion size. Constant contact produced the largest and intermittent contact the smallest lesions despite constant RF power and identical peak contact forces [11].

Catheter contact force can be evaluated by using 3 optical fibers to measure microdeformation of the catheter tip of an irrigated tip electrode (TactiCath, Endosense SA). It has an important impact on both ablation lesion size and the incidence of steam pops and thrombus incidence [12, 13].

Irrigated, flexible tip catheters as Therapy Cool Flex (St. Jude Medical, St. Paul, MA, USA) are designed to conform to the cardiac anatomy and thereby increase the tip-to-tissue interface. Such flexible tip catheters are also thought to reduce operator-transmitted force into tissue. Although clinical data with flexible tip catheter ablation of atrial fibrillation have been obtained (NCT01185613), no data for VT ablation are available yet. Irrigated tip catheters as ThermoCool Catheter (Biosense Webster, Diamond Bar, CA, USA) use a flexible catheter shaft design in order to improve tissue contact. The ThermoCool SmartTouch (Biosense Webster, Diamond Bar, CA, USA) catheter enables measurement of contact force and direction of force, using three sensors within the catheter shaft and the degree of spring bending between the catheter tip and the shaft. The ThermoCool SmartTouch Catheter has shown its efficacy for catheter ablation of paroxysmal AF [14]. This catheter was approved by the Food and Drug Administration (FDA) in February 2014 for the treatment of paroxysmal atrial fibrillation, sustained monomorphic ischemic VT, and atrial flutter and has been available in Europe since 2011.

An alternative technology to achieve more efficient intramyocardial lesions by improved catheter contact with target areas has recently been investigated. Patients with recurrent VT despite previous catheter ablation and antiarrhythmic drug therapy underwent ablation with the use of a new needle-tipped catheter. At target sites, the needle was advanced 7 to 9 mm into the myocardium, permitting pacing and recording as well as energy delivery through the needle. Injection of saline/iodinated contrast mixture ensured an intramyocardial position and excluded perforation. Further infusion was delivered before and during temperature-controlled RF ablation. By applying this technology control of some previously refractory VTs was feasible; however, complications were not rare [15].

## 4. Importance of Irrigation Rate

The irrigation of the catheter tip reduces excessive heating of the tissue and blood at the catheter tip and prevents the occurrence of thrombus and char formation. Due to the potential dramatic consequences, the prevention of thromboembolic events is of particular importance for left-sided procedures. As thrombus and char generate additional impedance and impair energy transfer to the tissue, the avoidance of thrombus and char formations would allow continuous energy transfer and lesion formation. The impact of different irrigation catheter flow on the development of lesion dimension and thrombus formation has been investigated in a thigh muscle preparation in sheep. RF ablations (30 s, 30 W) were performed with three different irrigation flow rates (5 mL/min, 10 mL/min, and 20 mL/min) and a perpendicular position with constant contact pressure of the irrigated ablation catheter (Sprinklr, Medtronic, Inc., Minneapolis, MN, USA). Cross sections of the lesions were investigated with regard to maximal depth and maximal diameter at and below the surface. During high flow irrigation (20 mL/min) the surface diameter was significantly smaller compared to irrigation flow rates of 5 mL/min and 10 mL/min. There was no significant difference in lesion depth with different flow rates. Thrombus formation was not observed during any RF application. Thus, lesion diameter does not increase with high irrigation flow rates [9]. Little irrigation seems to be sufficient to warrant effective ablation and to prevent thrombus and char formation. However, ablation with more than 30 W in anatomic locations with low blood stream might warrant higher flow rates than 20 mL/min and/or “intelligent” catheter design in order to prevent clot formation and to enable sufficient lesion size. Depending on catheter design and company recommendations power settings with up to 50 W at irrigation rates between 15 and 30 mL/min are used in clinical practice.

Irrigated, flexible tip catheters as Therapy Cool Flex (St. Jude Medical, St. Paul, MA, USA) have been designed to increase the contact surface and to improve the cooling effect. By directing up to 70% of the irrigation flow toward the tip-to-tissue interface the average tip temperature is approximately 5°C cooler in comparison to rigid tip catheters, with the possibility of reduced thrombus, char formation and steam pops. A larger number of irrigation channels are supposed to serve the same purpose. This concept has been successfully applied in the irrigated tip catheters ThermoCool SF Catheter (Biosense Webster, Diamond Bar, CA, USA). As mentioned above, no data for VT ablation are available yet.

## 5. Catheter Ablation of Ventricular Tachycardia after Myocardial Infarction

Compared with nonirrigated standard RF, open- and closed-loop irrigated RF catheter ablation proved to be more efficient in terminating VT in patients with prior myocardial infarction, a lower VT recurrence rate, and therefore facilitating successful ablation. The greater efficacy of cooled RF is consistent with the production of a larger lesion in human infarctions [16]. The efficacy of irrigated ablation technology for ablation of VT in remote myocardial infarction has also been investigated in a prospective multicenter trial, enrolling 63 patients (89% males) with a mean of 3 VTs. Ablation was acutely successful in 51 patients (81%). The results of this multicenter study demonstrate the high acute success rate and a low complication rate of irrigated tip catheter ablation of all clinical relevant VTs in remote myocardial infarction. However, during the follow-up a relevant number of recurrences occurred [17]. Similar to postinfarction VT, PVC after remote myocardial infarction most often originate within scar tissue and can be treated with catheter ablation at a high-success rate [18].

## 6. Epicardial Ablation

In ischemic and nonischemic cardiomyopathies as dilative cardiomyopathy (DCM) or arrhythmogenic right ventricular cardiomyopathy (ARVC) epicardial myocardium and mid-layer myocardium are often part of the arrhythmogenic tissue [19, 20]. Recent studies have shown that a combined endocardial-epicardial approach can be superior to an endocardial approach [21]. In contrast to endocardial ablation, lack of blood flow inside the epicardial space leads to a lack of convective cooling. Therefore, active cooling by catheter tip irrigation is used to ensure sufficient lesion size and depth. Due to anatomical reasons, catheter-tissue interface and contact force seem to be less in the epicardium than in the endocardium [20, 22]. Continuous aspiration of irrigation fluid during epicardial ablation prevents impedance loss and thus loss of RF energy to pericardial effusion [20].

## 7. Bipolar versus Unipolar Ablation

Ablation of ventricular tachycardia originating from the left ventricular epicardium, the interventricular septum, and the papillary muscles is often limited by the radiofrequency power delivery. RF ablation of VT from the LV papillary muscles is particularly challenging because they might be located relatively deep beneath the endocardium of the papillary muscles, which have a complex structure. Apart from applying more energy, bipolar ablation with higher density of RF current compared to unipolar ablation might be advantageous. With bipolar ablation, RF current flows between two catheter electrodes. In contrast, with unipolar ablation, RF current flows between the tip of the ablation catheter electrode and the indifferent electrode of the ground patch.

The effect of bipolar versus unipolar epicardial ablation on lesion size was compared in an ex vivo animal model with excised pig hearts. Bipolar ablation was performed between a 4 mm saline irrigated tip electrode on the LV epicardium and an opposing 10 mm non-irrigated-tip electrode on the LV endocardium. Epicardial lesion width did not differ between unipolar and bipolar ablation, but lesion depth was significantly greater and transmural lesions were achieved more often with bipolar compared to unipolar ablation. It has been concluded that bipolar ablation of the LV free wall is highly effective at creating an appropriately deep epicardial lesion. Nevertheless, steam pop occurred more often with bipolar ablation [23]. Bipolar ablation produced also a narrower, deeper lesion than unipolar ablation across the interventricular septum of excised swine hearts [24].

## 8. Conclusion and Perspectives

RF ablation with irrigated tip catheters decreases the likelihood of thrombus and char formation and enables the creation of larger lesions. Due to the potential dramatic consequences, the prevention of thromboembolic events is of particular importance for left-sided procedures. Although acute success rates of VT ablation are satisfactory, recurrence rate is high. Apart from the progress of the underlying disease, reconduction and the lack of effective transmural lesions play a major role for VT recurrences.

Further medical and technological progress is therefore needed to improve results and safety of catheter ablation. Potential areas of further development are the improvement of imaging integration technology, using three-dimensional contact and noncontact mapping together with magnetic resonance imaging or intracardiac ultrasound for better substrate definition and resolution [25, 26], the introduction of bipolar, multipolar, and needle ablation techniques into clinical routine, and the use of alternative sources of energy.
